# 5-Bis-(2,6-difluoro-benzylidene) Cyclopentanone Acts as a Selective 11β-Hydroxysteroid Dehydrogenase one Inhibitor to Treat Diet-Induced Nonalcoholic Fatty Liver Disease in Mice

**DOI:** 10.3389/fphar.2021.594437

**Published:** 2021-04-12

**Authors:** Hongguo Guan, Yiyan Wang, Huitao Li, Qiqi Zhu, Xiaoheng Li, Guang Liang, Ren-Shan Ge

**Affiliations:** ^1^ Department of Pharmacy, Zhejiang Hospital, Hangzhou, China; ^2^ Department of Anesthesiology, The Second Affiliated Hospital and Yuying Children’s Hospital, Wenzhou Medical University, Wenzhou, China; ^3^ School of Pharmaceutical Sciences, Wenzhou Medical University, Wenzhou, China

**Keywords:** 11b-hydroxysteroid dehydrogenase 2, 11b-hydroxysteroid dehydrogenase 1 inhibitor, cyclopentanone derivatives, nonalcoholic fatty liver disease, insulin resisitance

## Abstract

**Background:** 11β-Hydroxysteroid dehydrogenase one is responsible for activating inert glucocorticoid cortisone into biologically active cortisol in humans and may be a novel target for the treatment of nonalcoholic fatty liver disease.

**Methods:** A series of benzylidene cyclopentanone derivatives were synthesized, and the selective inhibitory effects on rat, mouse and human 11β-hydroxysteroid dehydrogenase one and two were screened. The most potent compound [5-bis-(2,6-difluoro-benzylidene)-cyclopentanone] (WZS08), was used to treat nonalcoholic fatty liver disease in mice fed a high-fat-diet for 100 days.

**Results:** WZS08 was the most potent inhibitor of rat, mouse, and human 11β-hydroxysteroid dehydrogenase 1, with half maximum inhibitory concentrations of 378.0, 244.1, and 621.1 nM, respectively, and it did not affect 11β-hydroxysteroid dehydrogenase two at 100 μM. When mice were fed WZS08 (1, 2, and 4 mg/kg) for 100 days, WZS08 significantly lowered the serum insulin levels and insulin index at 4 mg/kg. WZS08 significantly reduced the levels of serum triglycerides, cholesterol, low-density lipoprotein, and hepatic fat ratio at low concentration of 1 mg/kg. It down-regulated *Plin2* expression and up-regulated *Fabp4* expression at low concentration of 1 mg/kg. It significantly improved the morphology of the non-alcoholic fatty liver.

**Conclusion:** WZS08 selectively inhibits rat, mouse, and human 11β-hydroxysteroid dehydrogenase 1, and can treat non-alcoholic fatty liver disease in a mouse model.

## Introduction

Non-alcoholic fatty liver disease (NAFLD) is a common disease in humans and a liver manifestation of metabolic syndrome (Nakajima and Naito, 2015). It is closely related to our lifestyle and diseases, such as high-fat diet (HFD), type 2 diabetes, dyslipidemia, excessive adrenocortical hormone, obesity, and hypertension (Nakajima and Naito, 2015). Excessive consumption of HFD can lead to the accumulation of lipids in the liver, and is considered to be one of the main risks leading to NAFLD and a series of complications (Tsuchida et al., 2012; Softic et al., 2016), because of impaired liver lipid and glucose metabolism.

Some lipid-binding proteins may be associated with the progression of NAFLD. One family of these proteins is perilipins (PLINs). PLINs are associated with the surface of lipid droplets (Greenberg et al., 1991). Recent studies have shown that PLIN2 (encoded by *Plin2*) and PLIN3 (encoded by *Plin3*) are involved in the formation of lipid droplets and in the pathophysiological process of NAFLD, which is characterized by excessive accumulation of lipids in hepatocytes (Carr and Ahima, 2016; Graffmann et al., 2016; Sahini and Borlak, 2016).

Although the exact cause of NAFLD is unclear, the effects of hepatic glucocorticoid may be linked to metabolic syndrome and NAFLD. The glucocorticoid metabolizing enzyme 11β-hydroxysteroid dehydrogenase 1 (11β-HSD1, encoded by *Hsd11b1*) is considered to be related to NAFLD. 11β-HSD1 in fat and liver tissue converts inactive 11-ketoglucocorticoids [cortisone in humans or 11-dehydrocorticosterone (11DHC) in rodents] into biologically active 11β-hydroxy glucocorticoid cortisol or corticosterone (CORT) (Masuzaki et al., 2001; Morris et al., 2003). It has been found that overexpression of 11β-HSD1 in adipocytes in transgenic mice can cause visceral obesity and metabolic syndrome (Masuzaki et al., 2001). It was found that NAFLD in mouse models and human samples was associated with excessive portal cortisol and 11β-HSD1 overexpression in visceral fat (Candia et al., 2012). Indeed, a multicenter clinical trial using 11β-HSD1 inhibitors to treat NAFLD has demonstrated the effectiveness of reducing liver fat content, suggesting that 11β-HSD1 is a promising target (Stefan et al., 2014).

In addition to 11β-HSD1, there is another isoform, 11β-hydroxysteroid dehydrogenase 2 (11β-HSD2), which is mainly present in the kidney to regulate the role of mineralocorticoid receptors at pre-receptor level (White et al., 1994). 11β-HSD2 is a high-affinity enzyme that catalyzes the opposite reaction in activity by metabolizing cortisol to cortisone or CORT to 11DHC (Zhu et al., 2016). In the kidney, 11β-HSD2 acts as the gatekeeper for the mineralocorticoid receptor to exclude its binding to glucocorticoids because this receptor has the same affinity as the mineralocorticoid aldosterone or the glucocorticoid cortisol (White et al., 1994). 11β-HSD2 mutations in humans can cause apparent mineralocorticoid excess syndrome, such as hypertension and hypokalemia (White et al., 1997). Therefore, the purpose of this study was to develop a novel drug that targets 11β-HSD1 to treat NAFLD without affecting 11β-HSD2. In this study, a series of benzylidene cyclopentanone derivatives have been synthesized and evaluated to inhibit 11β-HSD1 and 11β-HSD2 activities. We have proved that 5-bis-(2,6-difluoro-benzylidene)-cyclopentanone (WZS08) is one of the most potent analogues that inhibit 11β-HSD1 without affecting 11β-HSD2, and can effectively treat HFD-induced NAFLD in mice.

## Materials and Methods

### Materials

High-fat diet (HFD) containing 10% fat, 20% sucrose, and 2.5% cholesterol (total energy content 329 KJ/100 g) was purchased from Beijing HFK Bioscience Co. (Beijing, China). Regular chow containing 3% fat, 10% sucrose, and 1% cholesterol (total energy content 167 KJ/100 g) was purchased from Zhejiang Experimental Animal Center (Hangzhou, China). Ultra-sensitive mouse insulin ELISA kit was obtained from CrystalChem (Elk Grove Village, IL). Oil Red O solution was purchased from Sigma-Aldrich (St. Louis, MO). Corticosterone ELISA kit was purchased from Abnova (Taiwan). [^3^H]-Cortisone, [^3^H]-CORT, and [^3^H]-cortisol were purchased from DuPont-New England Nuclear (Boston, MA) [^3^H]-11DHC was prepared from [^3^H]-CORT according to a previous method (Lakshmi and Monder, 1985). Cortisone, 11DHC, CORT, and cortisol were purchased from Steraloids (Newport, RI). Trypan blue (0.4%), reverse transcription agent with random primers, and Trizol kit were obtained from Invitrogen (Carlsbad, CA). SYBR Green qPCR kit and BCA Protein Assay kit were obtained from Takara (Otsu, Japan). Pierce ECL Western Blotting Substrate kit was purchased from ThermoFisher Scientific (Catalog 32,209; Waltham, MA). Radioimmunoprecipitation Assay buffer was obtained Bocai Biotechnology (Shanghai, China). Kidney and human liver microsomes were purchased from Gentest (Woburn, MA). Six-week-old male Kunming (KM) mice and four-week-old male Sprague Dawley rats were purchased from Shanghai Laboratory Animal Center (Shanghai, China). All animal procedures were approved by the Institutional Animal Care and Use Committee of Wenzhou Medical University and were performed in accordance with the Guide for the Care and Use of Laboratory Animals (Committee, 2011).

### Chemical Synthesis

The general procedure for the synthesis of benzylidene cyclopentanone derivatives was as follows: aromatic aldehyde (42.2 mmol) was added to a solution of 2.08 mmol cyclopentanone in ethanol (100 ml). The solution was stirred at room temperature for 10 min, and then NaOMe (54.8 mmol) solution was added dropwise. The mixture was stirred at a temperature of 25°C for 14 h. The mixture was poured into ice-water (150 ml) and filtered. The filtered cake was washed with water (20 ml × 2) and evaporated to dryness under reduced pressure. The solid part was purified by silica gel chromatography using CH_2_Cl_2_/CH_3_OH as an eluent. Thin-layer chromatography (TLC) was performed on Kieselgel 60 F254 plates. The melting point was measured on a Fisher-Johns melting apparatus and was uncorrected. ^1^H NMR spectrum was recorded on a Bruker 600 MHz instrument. The chemical shifts were presented in terms of parts per million using TMS as an internal reference. Electrospray ionization mass spectrometry (ESI-MS) data in positive mode was recorded on a Bruker Esquire 3000 spectrometer (Bruker, Billerica, MA). Column chromatography purification was performed on silica gel 60 (Merck, Kenilworth, NJ).

#### WZS01: (2E,5E)-2,5-bis(2,4-dichlorobenzylidene) Cyclopentanone

Yellow powder, 82.7% yield, mp 209.4–212°C. ^1^H-NMR (CDCl_3_) δ: 2.94 (s, 4H, CH_2_-CH_2_), 7.22–7.40 (m, 4H, Ar-H^5,6^ × 2), 7.45 (s, 2H, Ar-H^3^ × 2), 7.80 (s, 2H, Ar-CH = C × 2). ESI-MS *m/z*: 397.17 (M − 1)^+^, calculated for C_19_H_12_Cl_4_O: 398.11. Purity >95%.

#### WZS02: (2E,5E)-2,5-bis(2,4-dimethoxybenzylidene) Cyclopentanone

Yellow powder, 34.1% yield, mp 176.1–180.3°C. ^1^H-NMR (CDCl_3_) δ: 2.98 (s, 4H, CH_2_-CH_2_), 3.84 (s, 6H, Ar^4^-O-CH_3_ × 2), 3.86 (s, 6H, Ar^2^-O-CH_3_ × 2), 6.46 (s, 2H, Ar-H^3^ × 2), 6.52 (d, 2H, J = 8.0Hz, Ar-H^5^ × 2), 7.49 (d, 2H, J = 8. oHz, Ar-H^6^ × 2), 7.94 (s, 2H,Ar-CH = C × 2). ESI-MS *m/z*: 381.3 (M + 1)^+^, calculated for C_25_H_28_O_7_: 380.43. Purity > 95%.

#### WZS03: (2E,5E)-2,5-bis(2,4-dimethylbenzylidene) Cyclopentanone

Yellow powder, 72.6% yield, mp 128.8–131.8°C. ^1^H-NMR (CDCl_3_) δ:2.35 (s, 6H, Ar^4^-CH3 × 2), 2.43 (s, 6H, Ar^2^-CH3 × 2), 2.99 (s, 4H, CH_2_-CH_2_), 7.04–7.07 (m, 4H, Ar-H^3,5^ × 2), 7.40 (d, 2H, J = 8.0Hz, Ar-H^6^ × 2), 7.79 (s, 2H, Ar-CH = C × 2). ESI-MS *m/z*: 317.4 (M + 1)^+^, 339.3 (M + Na) calculated for C_25_H_28_O_7_: 316.44. Purity > 95%.

#### WZS04: (2E,5E)-2,5-bis(2,3-dichlorobenzylidene) Cyclopentanone

Yellow powder, 82.6% yield, mp 204.6–206.8°C. ^1^H-NMR (CDCl_3_) δ: 2.95 (s, 4H, CH_2_-CH_2_), 7.25 (t, 2H, J = 8.0Hz, Ar-H^5^ × 2), 7.41 (d, 2H, J = 8.0Hz, Ar-H^4^ × 2), 7.47 (d, 2H, J = 8.0Hz, Ar-H^3^ × 2), 7.88 (s, 2H, Ar-CH = C × 2). ESI-MS *m/z*: 399.23 (M + 1)^+^, calculated for C_25_H_28_O_7_: 398.11. Purity > 95%.

#### WZS05: (2E,5E)-2,5-bis(2,4,6-Trimethoxybenzylidene) Cyclopentanone

Yellow powder, 24.0% yield, mp 195–197.8°C. ^1^H-NMR (CDCl_3_) δ: 2.51 (s, 4H, CH_2_-CH_2_), 3.78 (d, 12H, J = 12.0Hz, Ar^2,6^-O-CH_3_ × 2), 3.82 (d, 6H, J = 12.0Hz, Ar^4^-O-CH_3_ × 2), 6.12 (s, 4H, Ar-H^3,5^ × 2), 7.55 (s, 2H, Ar-CH = C × 2). ESI-MS *m/z*: 441.16 (M + 1)^+^, calculated for C_25_H_28_O_7_: 440.49. Purity > 95%.

#### WZS06: (2E,5E)-2,5-bis(2-Carbolic Benzylidene) Cyclopentanone

Yellow powder, 77.7% yield, mp＞260°C [lit (Liang et al., 2008b).]. ESI-MS *m/z*: 345.3 (M − 1)^+^, calcd for C_24_H_26_O_7_: 346.38. Purity > 95%.

#### WZS07: (2E,5E)-2,5-bis(2,5-difluorobenzylidene) Cyclopentanone

Yellow powder, 29.4% yield, mp 208.1–209.8°C. ^1^H-NMR (CDCl_3_) δ: 3.05 (s, 4H, CH_2_-CH_2_), 7.06–7.10 (m, 6H, Ar-H), 7.73 (s, 2H, Ar-CH = C × 2). ESI-MS *m/z*: 687.3 (2M + Na)^+^, calculated for C_25_H_28_O_7_: 332.29. Purity > 95%.

#### WZS08: (2E,5E)-2,5-bis(2,6-difluorobenzylidene) Cyclopentanone

Yellow powder, 79.2% yield, mp 146.8149.6°C. ^1^H-NMR (CDCl_3_) δ: 2.73 (s, 4H, CH_2_-CH_2_), 6.92–6.95 (m, 4H, Ar-H^3,5^ × 2), 7.29–7.34 (m, 2H, Ar-H^4^), 7.50 (s, 2H, Ar-CH = C × 2). ESI-MS *m/z*: 334.4 (M + 1)^+^, 687.4 (M + Na), calculated for C_25_H_28_O_7_: 332.29. Purity > 95%.

#### WZ09: (2E,5E)-2,5-Bis(2-Hydroxy-3-Methoxylbenzylidene) Cyclopentanone

Green powder, 61.2% yield, mp 120.1e122.7C. ^1^H-NMR (CDCl_3_) δ: 7.74 (2H, s, Are-CH = C × 2), 7.19 (2H, m, Ar-H^6^ × 2), 7.13 (2H, m, Ar-H^5^ × 2), 6.98 (2H, m, Ar-H^4^ × 2), 5.35 (2H, s, Ar-OH × 2), 3.96 × (6H, s, Ar-O-CH_3_ × 2), 2.78 (4H, s, CH2-CH2). ESI-MS m/z: 351.2 (M − H)^−^, calculated for C22H14F8O: 352.38. Purity > 95%.

#### WZS10: (2E,5E)-2,5-Bis(2-Fluoro-3-(Trifluoromethyl)benzylidene) Cyclopentanone

Yellow powder, 58.1% yield, mp 221.8e222.4°C [223°C, lit (Liang et al., 2008b)]. ESI-MS m/z: 433.1 (M + H)^+^, calculated for C21H12F8O: 432.31. Purity > 95%.

### Preparation of Rat and Mouse Liver and Kidney Microsomes

The liver and kidney microsomes were prepared as previously described (Guo et al., 2012). Briefly, the tissue was homogenized in 0.01 mM phosphate buffered saline (PBS) containing 0.25 M sucrose, and the nuclei and large cell debris were removed by centrifugation at 1500 g for 10 min. The supernatant was transferred to a tube and the mitochondria were removed by centrifugation at 10, 000 × g for 30 min. The supernatant was transferred to an ultracentrifuge tube and centrifuged twice at 105,000 × g, the resulting microsomal precipitate was collected. The protein content was measured by the BCA kit according to the manufacturer’s instruction. The protein concentration was adjusted to 20 mg/ml. These proteins were used to measure 11β-HSD1 and 11β-HSD2 activities.

### Isolation of Rat Adult Leydig Cells

Leydig cells were isolated as previously described (Ge et al., 2006). Briefly, after a week of adjustment, eighteen 35-day-old Sprague Dawley rats were killed by asphyxiation with carbon dioxide (CO_2_). The testis was removed. The testis was perfused with collagenase solution through the testicular artery. The testis was digested with collagenase and DNase for 15 min, and the digested cells were filtered with nylon mesh, and the cells were separated under a Percoll gradient. The cells with density of 1.070–1.088 g/ml were collected and washed. The purity of the Leydig cell fraction was evaluated by histochemical staining of 3β-hydroxysteroid dehydrogenase using 0.4 mM etiocholanolone as a steroid substrate and 2 mM NAD + as a cofactor and tetranitroblue tetrazolium as the H^+^ acceptor as previously described (Payne et al., 1980). More than 95% of Leydig cells are strongly stained. Cell viability was estimated by measuring the percentage of cells that excluded trypan blue solution as previously described (Chen et al., 2007). In brief, an aliquot of 10^5^ cells/ml in a 0.5 ml test tube was mixed with 0.1 ml of 0.4% trypan blue at room temperature for 5 min and the cells were then loaded into a hemocytometer for counting, and the numbers of non-viable (staining) and viable (not stained) cells were counted. Viability was calculated as the percentage of viable cells divided by the total number of cells, and the viability of Leydig cells (intact Leydig cells) was over 99%. After incubation with chemicals, intact Leydig cells were used for the measurement of 11β-HSD1 activity.

### Determination of 11β-HSD1 in Rat, Mouse, and Human Liver Microsomes

The reductive activity of 11β-HSD1 in rat, mouse, and human liver microsomes was measured as previously described (Ge et al., 2005). In short, the 11β-HSD1 activity assay tube contained 25 nM substrate, 11DHC (for rats or mice) or cortisone (for humans), spiked with 60,000 dpm of each 3H-steroid. 11β-HSD1 activity was measured using 11-DHC or cortisone as a substrate. A 25 nM steroid substrate is used because the concentration is within the physiological concentration range. Rat (10 μg), mouse (10 μg) and human (4 μg) liver microsomes were incubated with 11-ketosteroids, 0.2 mM NADPH, and various concentrations (10^−10^–10^−5^ M) of drug candidates at 37 °C for 60–90 min. The inhibitory ability of the chemical relative to the control (DMSO solvent) was measured. The chemical was dissolved in DMSO to a final concentration of 0.4%, at which DMSO did not inhibit enzyme activity. The reaction was stopped by adding 10 µL of 1 mM glycyrrhetinic acid and 1 ml of ice-cold ether. The steroid was extracted with ether, and the organic layer was dried under nitrogen. The steroids in chloroform and methanol (90:10, v/v) were chromatographically separated on a thin layer plate, and radioactivity was measured using a scanning radiometer (System AR2000, Bioscan Inc., Washington, DC, United States). The percentage of 11DHC being converted to CORT or cortisone being converted to cortisol can be calculated by dividing the radioactivity count of 11-OH-steroids by the total count. 11β-HSD1 is abundantly expressed in the livers of all species.

### Measurement of 11β-HSD1 in Intact Rat Leydig Cells

To test whether chemicals can penetrate cell membranes to inhibit 11β-HSD1 activity, intact rat Leydig cells were used because this cell type contains the highest level of 11β-HSD1 of all cell types (Monder et al., 1994). The reductive activity of 11β-HSD1 in rat Leydig cells was measured as previously described (Ge et al., 1997). Briefly, the 11β-HSD1 activity assay tube contained 25 nM substrate, of which 11DHC was spiked with 60,000 dpm of each 3H-11DHC without the addition of NADPH. The reaction was initiated after adding 0.1 ×10^6^ intact Leydig cells. Other procedures were described above.

### Measurement of 11β-HSD2 Activity in Rat, Mouse, and Human Kidney Microsomes

The 11β-HSD2 assay was based on rat, mouse, and human kidney microsomes, as previously described (Ge et al., 2005). In brief, the 11β-HSD2 activity assay tube contained 25 nM substrate (cortisol for humans, CORT for rats and mice). Renal microsomes (10 μg) were incubated with substrate and 0.2 mM NAD^+^ for 30 min. Other procedures were described above. The percentage conversion of cortisol to cortisone (humans) or CORT to 11DHC (rats and mice) was calculated by dividing the radioactive count of an 11keto-steroid product by the total count associated with both substrate and product.

### Determination of Half Maximum Inhibitory Concentrations (IC_50_)

The IC_50_ was determined by adding 25 nM steroid substrate with 0.2 mM cofactor and various concentrations (10 nM–100 μM) of WZS08 to 250 μL PBS (0.1 mm, pH = 7.2) containing 11β-HSD1 or 11β-HSD2 microsomal protein and each reaction mixture was incubated for 60 min. In addition, when Leydig cells were involved, 2000 nM of steroid substrate with 0.4 mM cofactor and various concentrations (10 nM–100 μM) of WZS08 was added to 500 μL of reaction buffer (0.1 mM phosphate buffer) to determine the IC_50_ value.

### Animal Treatment

The KM mice were randomly divided into six groups, including a regular chow (Group C), HFD (Group M) as a model control group, HFD supplemented with various doses of WZS08, 1 (Group M + W1), 2 (Group M + W2), and 4 (Group + W4) mg/kg, 20 mice per group. Mice were housed five animals per cage. Mice were maintained at a constant temperature (25 ± 3°C) for a 12-h light-dark cycle with free access to food and water. After a week of diet adaptation, the mice were fed with WZS08 dissolved in 1% sodium carboxymethyl cellulose (CMC-Na) or 1% CMC-Na for 100 days alone. Each mouse was gavaged daily with 0.5 ml vehicle (1% CMC-Na) or drug in 1%CMC-Na. By the end of the treatment, the mice were killed by CO2, and the weights of body, liver, epididymal fat, mesenteric fat, and dorsal fat were measured.

### Oral Glucose Tolerance Test

Oral glucose tolerance test was performed according to a previously described method with some modifications (Horakova et al., 2016). The oral glucose tolerance test is considered to be a method for analyzing the homeostasis of circulating glucose in mice. In brief, at the end of the second week, the mice were fasted for 6 h (7:00 am1:00 pm) and blood samples were collected. Insulin levels were measured by commercial ELISA analysis kit according to the manufacturer’s instructions. After oral administration of glucose, the blood glucose meter (Sinocare Inc., Changsha, China) was immediately used to measure the glucose level from the tail blood before 0 (in rapid state) or after 15, 30, 60, and 120 min.

### Intraperitoneal Insulin Sensitivity Test

The intraperitoneal insulin sensitivity test was performed at the last week, mice were fasted 4 h and then administered insulin intraperitoneally (0.75 IU/kg, Eli Lilly, IN) as previously described (Prasad Sakamuri et al., 2012; Ma et al., 2016). At 0, 15, 30, 60, and 120 min, tail blood was collected and the glucose levels were measured using a blood glucose meter as described above.

### The Calorie of Consumed Food

The energy intake of the mice in each experimental group is different. The weights of the HFD and regular chow consumed by mice in the experimental group were calculated within the next 45 days after drug treatment. HFD contained an energy of 329 KJ/100 g and regular chow contained an energy of 167 KJ/100 g. The calories consumed by mice in each experimental group during the 45 days was calculated.

### Extraction of Lipids From Feces and Liver

The extraction of fecal and liver lipids was performed according to the previously published method with only minor modification (Morton et al., 2005; Zhao et al., 2015). Briefly, the feces were collected during a 24 h period at the last week, then these samples were immediately frozen at −80°C. Total lipids were extracted from 100 mg dried feces that were cleaned by picking, carefully chosen using tweezers, and dried for 12 h by a freeze-dryer, and 300 mg fresh liver that was cut into small pieces, respectively. Then, these samples were individually incubated with 2–6 ml of chloroform/methanol (2:1, v/v) for 30 min at 60°C with continuous stirring for 30 min. After the incubation, the mixture was centrifuged at 500 ×*g* for 5 min, and the supernatant was collected. An aliquot of water (1 ml) was added to the supernatant, and the mixture was vortexed and centrifuged at 500 ×*g* for 10 min. The lower chloroform layer was transferred to a pre-weighed tube, and the sample was dried by a nitrogen evaporator and weighed.

### Oil Red-O Staining

Oil red-O staining was performed as previously described (Hunt et al., 2013; Legeza et al., 2014). Briefly, liver samples were freshly collected and frozen in liquid nitrogen. Frozen sections were cut to a thickness of 8 μm under a cryostat. The sections were then stained with 0.5% oil red-o solution for 10 min. The sections were counterstained with hematoxylin and washed. Sections were visualized by Leica microscope and photographs were taken.

### Quantitative Real-Time Polymerase Chain Reaction

Total RNA was isolated from mouse liver using Trizol kit according to the manufacturer’s instruction. To reduce genomic DNA contamination, total RNAs were treated with ribonuclease-free deoxyribonuclease I (Thermo Fisher Scientific, Waltham, MA, United States) for 1 h at 37°C. 1 μg RNA was used to synthesize the first strand cDNA by reverse transcription kit. qPCR was carried out using SYBR GREEN as an indicator using Bio-Rad real time PCR system. The standard curve method was adopted as previously described (Lv et al., 2019). The Ct values were recorded after a series of diluted standards of mouse liver cDNAs, and the linear equation of Ct values vs. logarithm [dilution of standards] was generated, and the concentration of the target mRNA was calculated according to their Ct value. The result was normalized to *Gapdh* (encoding GAPDH) and *Rps16* as the internal controls. The mRNA levels of *Acs2* (encoding acetyl-CoA synthetase2), *Hsd11b1* (encoding 11β–HSD1), *Ldlr* (encoding LDLR), *Plin2* (encoding PLIN2), and *Plin3* (encoding PLIN3) were evaluated. There were no changes in *Gapdh* and *Rps16* mRNA levels between groups and the test mRNA levels were adjusted to *Gapdh*. Primer information is listed in Supplementary Table S1.

### Measurement of Serum Insulin and Corticosterone Levels and Blood Lipid Levels

On the last day of the experiment, the mice were fasted for 6 h (with free access to drinking water), and then blood and tissues were sampled. Blood samples were collected. The blood sample was centrifuged at 5000 rpm for 10 min to collect the serum, and the serum was frozen at -40°C until use. According to standard clinical protocols, the levels of serum glucose, low-density lipoprotein (LDL), high-density lipoprotein (HDL), total cholesterol, and triglyceride (TG) were measured using Hitachi 7600 biochemical analyzer (Hitachi, Japan). In addition, serum insulin and corticosterone levels were evaluated using the ultrasensitive mouse insulin ELISA kit (CrystalChem, IL, USA) and the commercially available corticosterone ELISA kit (Abnova, Taiwan, China) according to the manufacturers’ instructions.

### Protein Expression Analysis

Total hepatic protein extract was obtained as previously described (Lazaro et al., 2013). The protein was separated by SDS–PAGE and transferred to a 0.2 μm PVDF membrane. The primary antibodies used were: 11β-HSD1 (rabbit polyclonal antibody, Abcam, catalog # ab39364; being blocked with 11β-HSD1 peptide, #ab101097, San Francisco, CA), ACS2 (Abcam, catalog # ab229958, San Francisco, CA, United States), and β-actin (ACTB, Cell signaling, Danvers, MA, United States). Goat anti-rabbit HRP was used as a secondary antibody. Finally, chemiluminescence emitted by the immunoreactive protein was detected by an ECL advance agent, and visualized with a MicroChemi Imaging system and qualified by a densitometry using AlphaEaseFC (ProteinSample, CA, United States). Relative protein levels were normalized by ACTB.

### Histological Examination

Before hematoxylin and eosin (HE) staining, the livers of KM mice were fixed in 10% formalin, dehydrated, and embedded in paraffin, as previously described (Andres-Manzano et al., 2015). In brief, the sample was cut to 6 μm sections. Paraffin sections were dewaxed, and hydrated under gradient ethanol and stained with HE staining solution. Then, the sections were dehydrated in gradient ethanol and xylene and mounted with neutral resin.

### Statistical Analysis

The data are expressed as mean ± SEM (standard errors). A mixed multiple regression model for repeated measurement was used to compare body weight, plasma insulin, and glucose resistance by SPSS (version 18.0, IBM Inc., Chicago, IL). In additional to data that was mentioned above, the other results were subjected to two–way ANOVA and then *post hoc* Turkey’s test for diet and drug using GraphPad (Version 6, GraphPad Software Inc., San Diego, CA, United States). Data in untreated HFD mice was compared to the results in STD control mice; data in treated HFD mice were compared to the results in untreated HFD mice. Statistical significance was set at *p* < 0.05.

## Results

### Chemistry

**FIGURE 1:**
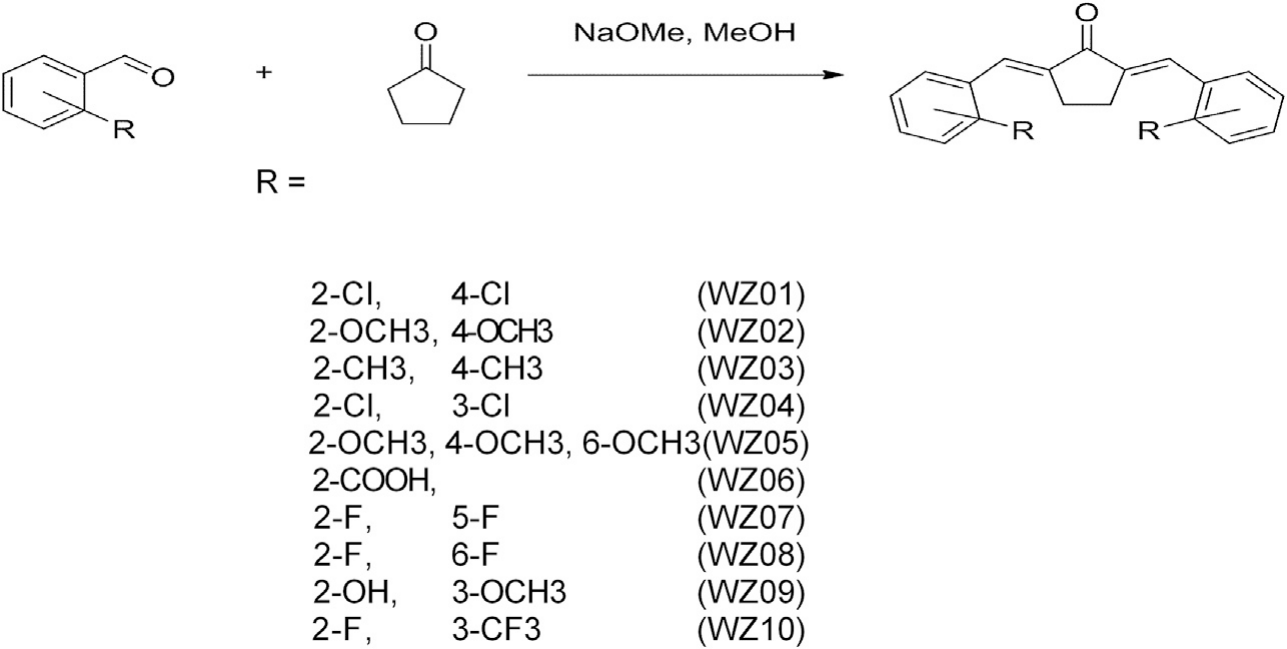
Synthetic method and chemical structures of benzylidene cyclopentanone derivatives. Ten compounds (WZS01-WZS10) are listed.

According to our previous report (Figure 1) (Liang et al., 2008a), a series of benzylidene cyclopentanone derivatives were synthesized using appropriate aromatic aldehydes and cyclopentanone under basic conditions. When different substituents with vivid electronic properties were substituted in the two benzene rings, the structure-activity relationship of these compounds was analyzed. In addition, the production process, purity, H3 NMR analysis and other properties of these derivatives were recorded.

### Inhibition of 11β-HSD1 and 11β-HSD2 by Chemicals

**FIGURE 2:**
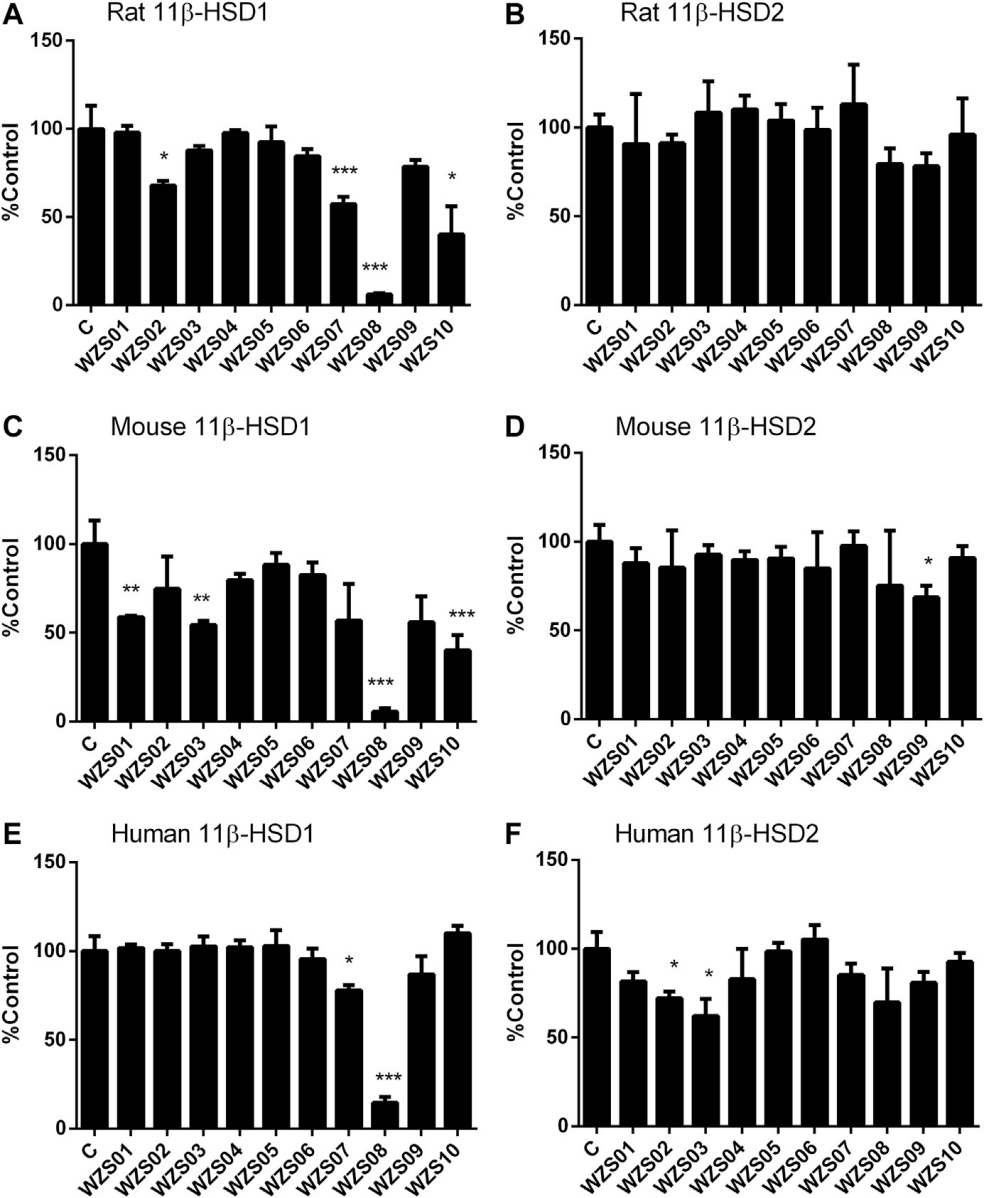
Effects of benzylidene cyclopentanone derivatives on rat, mouse, and human 11β-HSD1 and 11β-HSD2. Panel A–C: 11β-HSD1; Panels D–F: 11β-HSD2; Panels A and D: rat; Panels B and E: mouse; Panels C and F: human. Mean ± SEM, *n* = 4 (batches of assay). *, **, *** indicate significant differences at *p* < 0.05, *p* < 0.01*,* and *p* < 0.001, respectively, when compared to the control.

**FIGURE 3:**
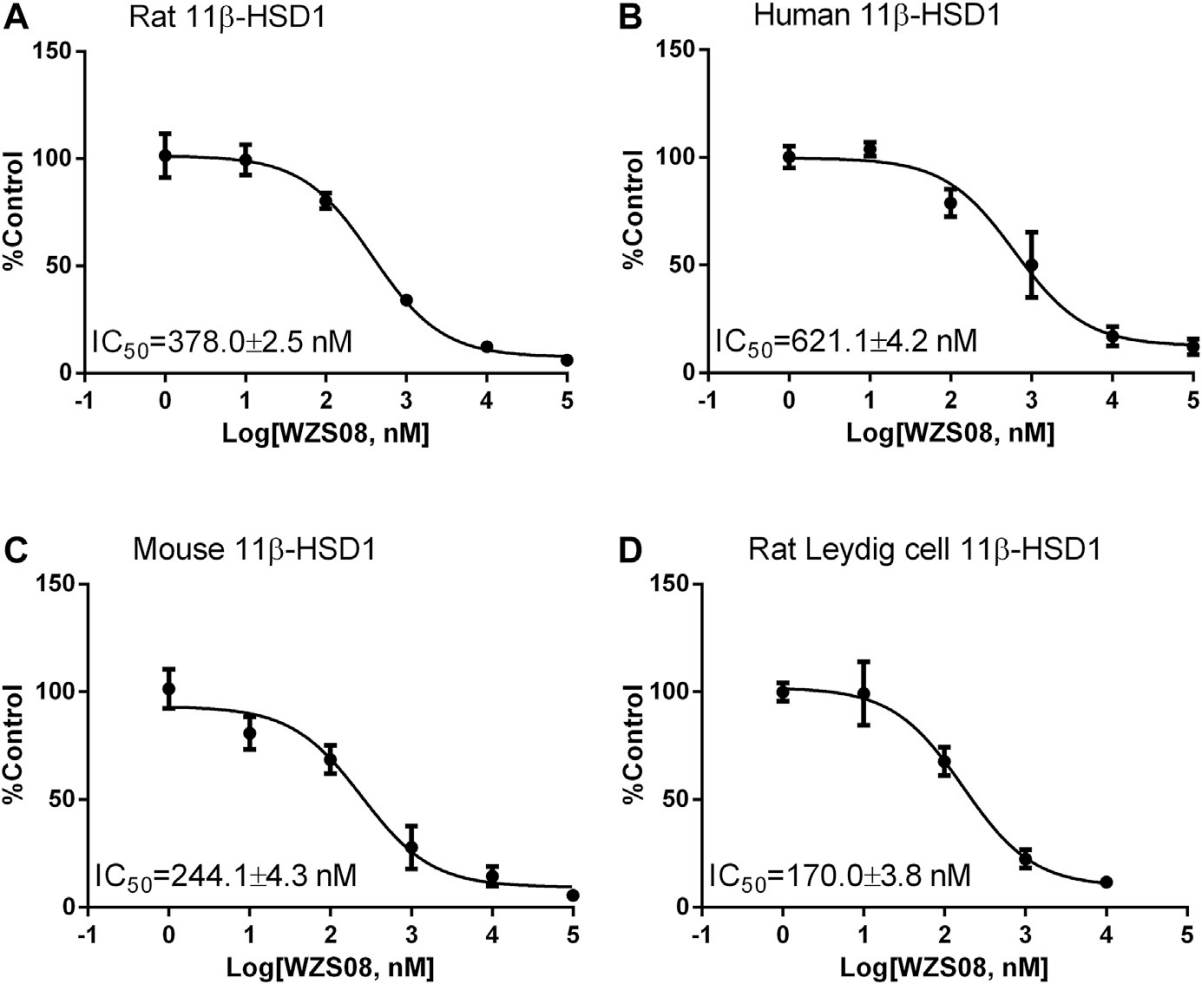
The half maximum inhibitory concentration (IC_50_) values of WZS08 on 11β-HSD1 activities in rat, mouse, and human liver microsomes and intact rat Leydig cells. Panel A, rat microsome; Panel B, mouse microsome; Panel C, human microsome; Panel D, intact rat Leydig cells. Mean ± SEM, *n* = 4 (batches of assay).

Previous studies have demonstrated that several similar chemicals are potent and selective 11β-HSD1 inhibitors, and their inhibitory effects on human and rat enzymes were tested (Lin et al., 2013; Yuan et al., 2014). In this study, the efficacy of inhibiting 11β-HSD1 and 11β-HSD2 in three species (rat, mouse, and human) was performed. As shown in Figure 2 100 μM of each chemical was used for initial screening. Among all the chemicals tested, only WZS08 caused 11β-HSD1 inhibition to exceed 50% (with 93.90 ± 0.42%, 94.48 ± 0.97%, and 85.44 ± 1.67% inhibition for rat, mouse, and human enzymes, respectively), although the degree of inhibition of 11β-HSD1 by several other chemicals depended on the species. When the highest concentration (100 μM) was used, WZS08 had almost no effect on the 11β-HSD2 activity of all species. These results indicate that WZS08 is a selective inhibitor of 11β-HSD1. We further evaluated the IC_50_ value of WZS08 by inhibiting 11β-HSD1 activity in all three species, and we demonstrated that WZS08 is a potent inhibitor of 11β-HSD1 with IC_50_ in the nanomolar range (Figure 3). We tested whether WZS08 can penetrate cell membrane to inhibit 11β-HSD1 using intact rat Leydig cells because this cell type has the highest expression level of this enzyme in the rat (Monder et al., 1994). As shown in Figure 3D, we found that it can also potently inhibit rat 11β-HSD1 in intact cells.

### Body Weight, Fecal Lipids, and Calorie Intake

**FIGURE 4:**
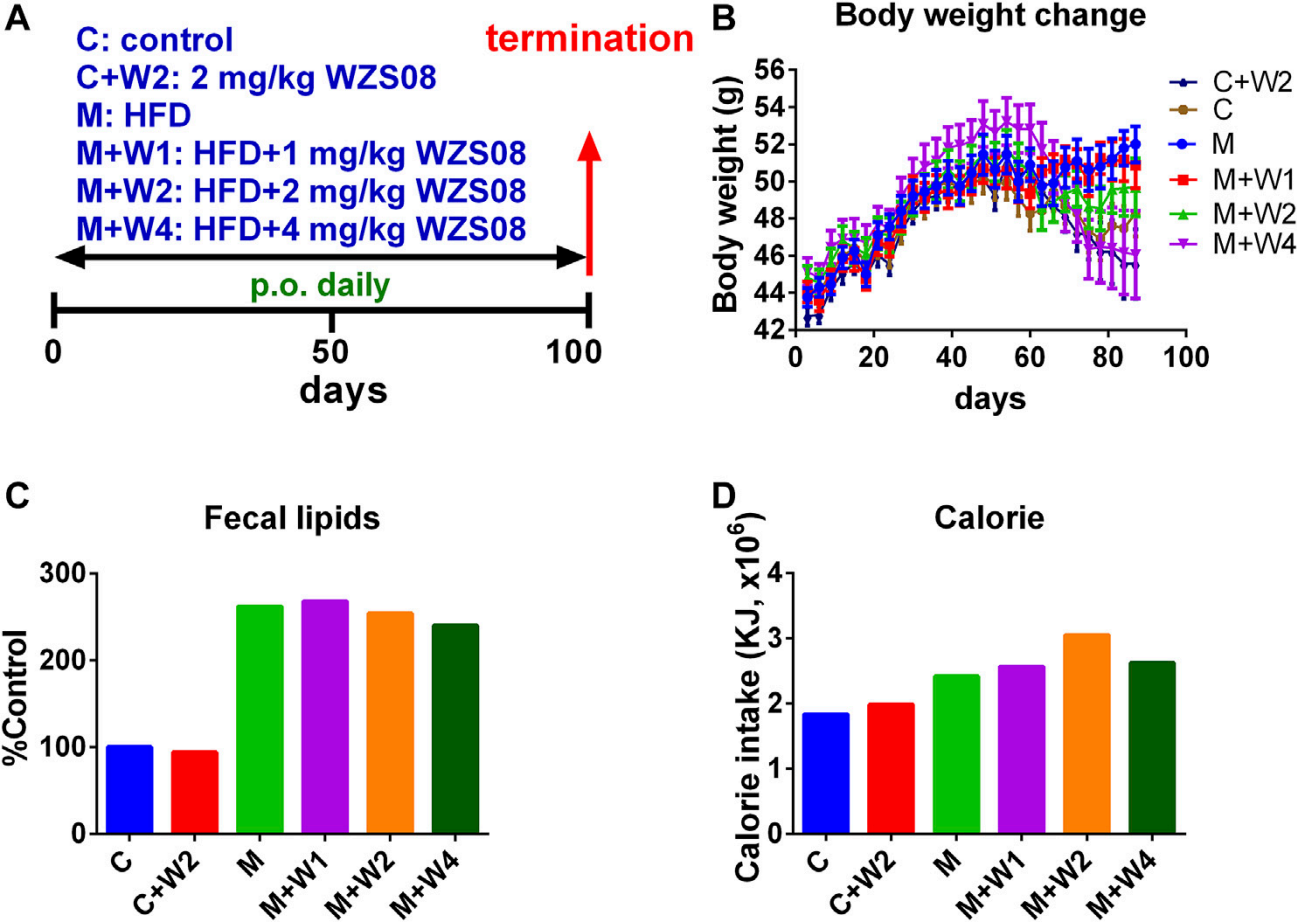
Regimen of animal study and the effect of WZS08 on body weight, fecal lipid content, and calorie intake. Panel A, regimen of treatment; Panel B, body weight of animals on different groups; Panel C, % fecal lipid weight of control group at the end of experiment; Panel D, calorie intake (in 45 days) was calculated after mice were fed for 2 weeks. Group **C**: the regular chow; Group C + W2: the regular chow plus 2 mg/kg WZS08; Group M: high fat diet (HFD); Group M + W1: HFD plus 1 mg/kg WZS08; Group M + W2: HFD plus 2 mg/kg WZS08; Group M + W3: HFD plus 4 mg/kg WZS08. Mean ± SEM, n = 20 (animals).

We evaluated WZS08 in the HFD-induced NAFLD mouse model (Figure 4A). At the end of the study (100 days after drug treatment), the body weight of HFD (Group M) was significantly higher than that of the control group (Group C, Figure 4B). Compared with the HFD control, WZS08 dose-dependently reduced body weight changes (Figure 4B). We found that the total fecal lipid content of Group M was higher than that of control group (Group C) (Figure 4C). Compared with Group M, treatment with WZS08 did not affect the total fecal lipid content (Figure 3B), indicating that WZS08 did not affect lipid absorption. The calorie intake of the HFD control group (Group M) was higher than that of the regular chow control group (Group C, Figure 4D). Compared with Group M, treatment with WZS08 did not affect calorie intake (Figure 4D), indicating that WZS08 did not affect appetite.

### Effect of WZS08 on Tissue and Organ Weights

After the treatment, we measured the weight of liver, back fat, visceral fat, epididymal fat, and thymus. Weight was adjusted by the length of the tibia, which was more tightly associated with the age of days than the body weight (Duyar and Pelin, 2003). Compared with the control group (Group C), HFD increased the weight of the liver, and compared with the HFD group (group M), WZS08 dose-dependently reduced the weight of the liver. The weight of other tissues was not affected (Table 1).

**TABLE 1:** The weights of tissues and organs.

Tissue and Organ	C	C + W2	M	M + W1	M + W2	M + W4
Body weight (g)	51.76 ± 1.22	50.70 ± 0.92	51.36 ± 1.31	52.46 ± 1.28	50.25 ± 1.01	49.00 ± 1.22
Total hepatic weight (g)	2.08 ± 0.08	2.03 ± 0.06	2.47 ± 0.08****	2.79 ± 0.12****	2.48 ± 0.06****	2.29 ± 0.06*
Thymic adipose weight (g)	0.07 ± 0.01	0.07 ± 0.01	0.068 ± 0.00	0.08 ± 0.01	0.06 ± 0.00	0.077 ± 0.00
Dorsal adipose weight (g)	0.55 ± 0.068	0.49 ± 0.045	0.51 ± 0.063	0.68 ± 0.092	0.54 ± 0.049	0.55 ± 0.070
Mesenteric adipose weight (g)	0.64 ± 0.059	0.55 ± 0.044	0.49 ± 0.053	0.64 ± 0.074	0.55 ± 0.043	0.54 ± 0.048
Epididymal adipose weight (g)	1.51 ± 0.154	1.30 ± 0.116	1.64 ± 0.222	2.05 ± 0.230	1.52 ± 0.138	1.54 ± 0.20
Tibial length (cm)	2.45 ± 0.060	2.34 ± 0.046	2.31 ± 0.051	2.51 ± 0.053	2.41 ± 0.056	2.45 ± 0.071

The data were collected 100 days after drug treatment.

### Effect of WZS08 on Insulin Homeostasis and Lipid Distribution

**FIGURE 5:**
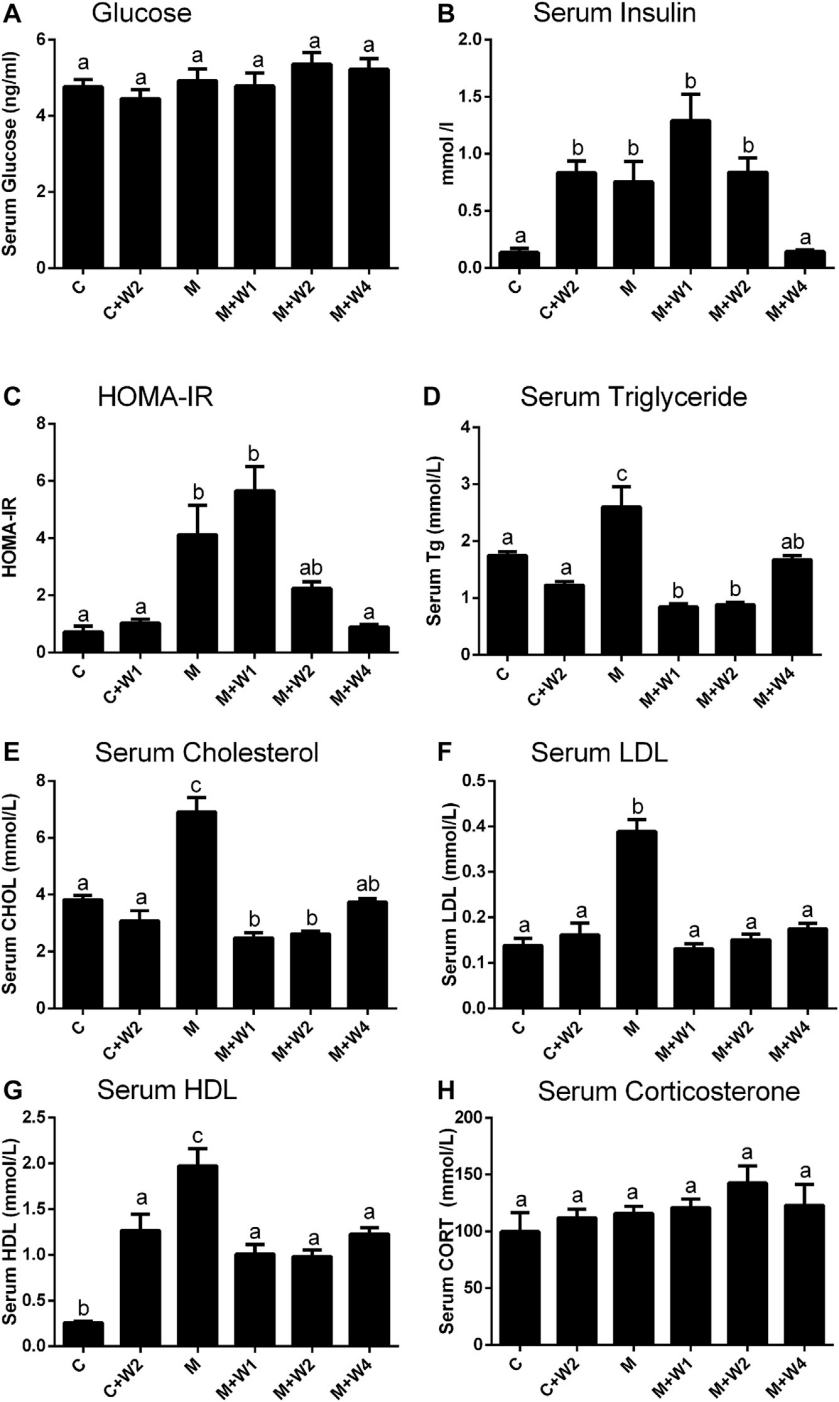
Effects of WZS08 on plasma glucose and lipid parameters. Serum was collected at the end of experiment in the fast state. Panel A, glucose; Panel B, serum insulin; Panel C, HOMA-IR; Panel D, serum TG **(D)**; Panel E, serum cholesterol; Panel F, serum LDL **(F)**; Panel G, serum HDL. HOMA-IR was obtained by formula: HOMA-IR = Insulin (mUI/L) × serum glucose (mmol/L)/22.4. Mean ± SEM, *n* = 20 (animals). There are significant differences between the two groups with different alphabets (*p* < 0.05).

Previous studies have reported that long-term HFD-fed animals developed insulin resistance (Gallou-Kabani et al., 2007). Although there was no difference in blood glucose levels between the groups, HFD (Group M) caused a significant increase in serum insulin levels. High dose (Group M + W4, 4 mg/kg) of WZS08 completely reduced it to control level (Figure 5B). The index of insulin resistance was estimated as HOMA-IR, showing that WZS08 dose-dependently reduced insulin resistance (Figure 5C). Blood lipids were considered to be the most important biomarker of NAFLD (Makovicky et al., 2014; Ichino et al., 2015). In this study, we evaluated lipid profile. Compared with controls, HFD (Group M) can increase serum triglyceride, cholesterol, LDL, and HDL levels. The high dose (4 mg/kg, Group M + W4) of WZS08 greatly reduced their levels (Figures 5D–G). Interestingly, WZS08 did not change the systemic circulatory CORT level (Figure 5H), indicating that it acts locally.

### Effect of WZS08 on Liver Lipids

**FIGURE 6:**
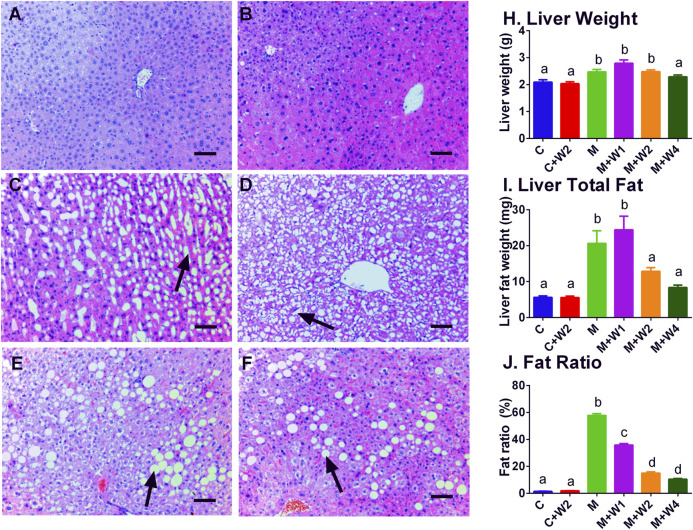
Effects of WZS08 on liver histochemical staining and lipid content. Panel **A–F**: HE staining for the regular chow (Group C), regular chow plus 2 mg/kg WZS08 (Group C + W2), high fat diet (HFD) (Group M), HFD +1 mg/kg WZS08 (Group M + W1), HFD +2 mg/kg WZS08 (Group M + W2), HFD+4 mg/kg WZS08 (Group M + W4), respectively. Arrow designates the lipid. Bar = 30 μm. Panel **G**: liver weight; Panel **H**, total liver lipids; Panel **I**, the fat ratio. Mean ± SEM, *n* = 20 (animals). There are significant differences between the two groups with different alphabets (*p* < 0.05).

**FIGURE 7:**
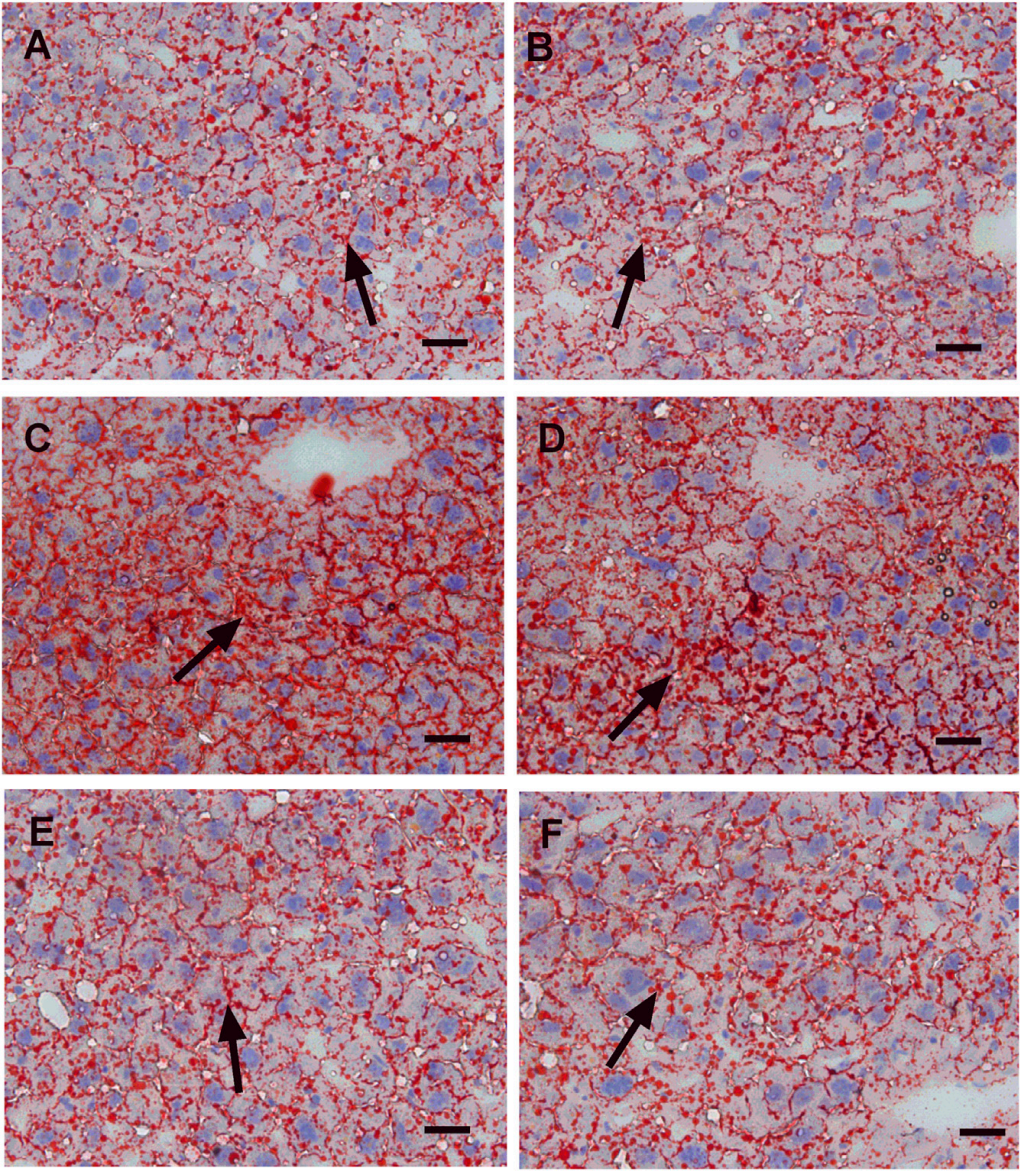
Oil red-o staining of liver sections. Panel **A–F**: HE staining for the regular chow (Group C), regular chow plus 2 mg/kg WZS08 (Group C + W2), high fat diet (HFD) (Group M), HFD +1 mg/kg WZS08 (Group M + W1), HFD +2 mg/kg WZS08 (Group M + W2), HFD+4 mg/kg WZS08 (Group M + W4), respectively. Arrow designates the fibrotic region. Bar = 20 μm.

In this study, we performed HE staining to study the effect of WZS08 on liver lipids. We found that the HFD-treated liver (Figure 6C) contained more lipid space than the regular chow control group (Figure 6A) and WZS08 (2 mg/kg) +chow group (Figure 6B). WZS08 remarkably reduced liver lipid content as shown in Figures 6D–F. The histochemical changes paralleled liver weight (Figure 6H), liver total lipid (Figure 6I), and lipid ratio (Figure 6J), indicating that WZS08 significantly reduces liver lipid content. We also conducted an oil-O staining to investigate the effects of WZS08 on lipids in the liver. Histochemistry with oil-O staining showed similar changes of liver lipids (Figure 7).

### Effect of WZS08 on the Expression of Lipid-Related Proteins

**FIGURE 8:**
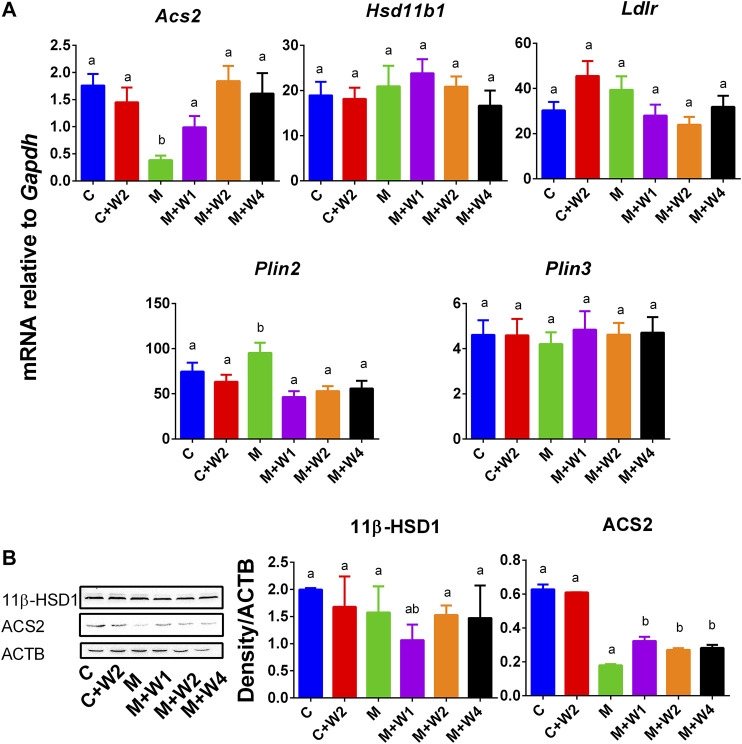
mRNA and protein levels and of lipogenesis and lipid metabolism related gene. Panel A, Acs2, *Ldlr*, *Hsd11b1*, *Plin2,* and *Plin3*. Mean ± SEM, n = 13 (randomly selected samples). Panel B, Western blot for 11β-HSD1 and ACS2 and the protein level of 11β-HSD1 and ACS2 relative to ACTB. Mean ± SEM, *n* = 3 (randomly selected samples). There are significant differences between the two groups with different alphabets (*p* < 0.05).

Previous studies have shown that PLIN2 was positively correlated with NAFLD (Orlicky et al., 2019) while ACS2 was negatively associated with NAFLD (Kim et al., 2004). We examined the expression of liver genes, including *Acs2*, *Ldlr*, *Hsd11b1*, *Plin2*, and *Plin3* (Figure 8). We found that the expression of *Acs2* (Figure 8A) in the HFD (M) group was significantly down-regulated, and compared with Group M, WZS08 (1, 2, and 4 mg/kg) up-regulated its levels (Figure 8A). In contrast, the expression of *Plin2* (Figure 8A) in the HFD (M) group was up-regulated, and compared with Group M, WZS08 (1, 2, and 4 mg/kg) down-regulated its levels. The expression of the other genes examined did not change (Figure 8A). Histochemical staining (Supplementary Figure S1) and Western blotting (Figures 8B,C) further confirmed that liver 11β-HSD1 was unchanged, indicating that WZS08 works by inhibiting 11β-HSD1 enzyme activity rather than by down-regulating its expression. However, ACS2 (acetyl-CoA synthetase 2) change paralleled with its mRNA level (Figure 8C).

## Discussion

We have previously reported that many mono-carbonyl curcumin analogues had inhibitory properties on human and rat 11β-HSD1 activity (Lin et al., 2013). One of series of these analogues is benzylidene cyclopentanone. We synthesized ten novel benzylidene cyclopentanone derivatives and evaluated their potencies to inhibit rat, mouse, and human 11β-HSD1 and judged their selectivity to 11β-HSD1 not to 11β-HSD2. We found that WZS08 is the most potent compound to inhibit 11β-HSD1 without affecting 11β-HSD2 in all three species.

Our previous study showed that 5-bis-(2-bromo-benzylidene) cyclopentanone, an analog of WZS08, had IC_50_ values of inhibiting rat and human 11β-HSD1 of 148 and 346 nM, respectively (Lin et al., 2013). In the current study, we demonstrated that WZS08 had similar potency to inhibit rat (IC_50_ of 378 nM) and human (IC_50_ of 621 nM) 11β-HSD1.

11β-HSD1 converts cortisone or 11DHC (inactive) to cortisol or CORT (active) in the liver and adipose tissue in humans or rodents (rats and mice), respectively (Tomlinson et al., 2004). Our data demonstrated that WZS08 potently inhibited 11β-HSD1 in all three species (rat, mouse, and human). This animal study also showed that the administration of WZS08 can improve metabolic dysfunction by inhibiting 11β-HSD1 activity in HFD-fed mice. The role of 11β-HSD1 in the pathogenesis of metabolic syndrome such as insulin resistance, hypertension, and glucose intolerance has been demonstrated in the transgenic mouse model, in which 11β-HSD1 was overexpressed in the adipose tissue (Masuzaki et al., 2001). Morton et al. demonstrated that mice with 11β-HSD1 deletion in adipose tissue were resistant to HFD-induced glucose intolerance (Morton et al., 2004), suggesting that 11β-HSD1 is a promising target for the treatment of metabolic syndrome and NAFLD. WZS08 significantly inhibited 11β-HSD1 to prevent HFD-induced insulin resistance, thus confirming the role of 11β-HSD1 in the pathogenesis of metabolic syndrome. Therefore, in this study, we further explored the role of WZS08 in preventing NALFD in the HFD-treated mouse model. NAFLD represents a series of conditions with histological features ranging from non-alcoholic steatohepatitis and fibrosis to hepatocellular carcinoma (Soares E Silva et al., 2015; Goh and McCullough, 2016).

WZS08 treatment for three months resulted in a significant decrease in the mean liver fat content of HFD-induced NAFLD mice, reaching the level similar to normal chow treatment (C group) by the highest dose (4 mg/kg). We found that at doses of 2 and 4 mg/kg, WZS08 significantly decreased liver fat content after inhibiting 11β-HSD1 (Figure 6). We examined liver sections to explore whether 11β-HSD1 inhibition has an improved effect on liver histology in HFD-induced NAFLD mice. Indeed, the liver histochemistry after WZS08 treatment was significantly improved, as shown by HE (Figure 6) and Oil Red-stain staining (Figure 7).

Although blood glucose in HFD-treated mice did not show significant changes by oral administration of WZS08, the result indicates that WZS08 improved glucose tolerance and insulin sensitivity as judged by serum insulin levels (Figure 5B) and HOMA-IR (Figure 5C). WZS08 also reduced the levels of liver triglycerides (Figure 5D), cholesterol (Figure 5E) and LDL (Figure 5F) levels. It is known that defects in lipid metabolism and insulin resistance are manifestations of metabolic syndrome. Interestingly, there was no change in serum CORT levels, indicating that inhibition of 11β-HSD1 resulted in a reduction in local CORT levels rather than a reduction in systemic levels. Indeed, the metabolic syndrome is mainly caused by the local activation of glucocorticoids in the liver and adipose tissue (Masuzaki et al., 2001).

The immune response has been shown to be involved in the pathogenesis of NAFLD. Local signals related to microbial balance, bacterial translocation or signals derived from adipose tissue or the intestinal tract activate immune cells and recruit more immune cells into the liver. These effects result in inflammatory responses, which may lead to cell damage and death, thereby promoting NAFLD disease progression (Arrese et al., 2016). It is well known that 11β-HSD1 influences immune responses. During inflammation, 11β-HSD1 has been reported to be upregulated. 11β-HSD1-deficient mice exhibited delayed acquisition of macrophage phagocytic capacity, whereas cytokine production in macrophages lacking 11β-HSD1 was increased (Zhang et al., 2005; Gilmour et al., 2006; Coutinho et al., 2016; Valbuena Perez et al., 2020). Thus, the systemic inhibition of 11β-HSD1 increase inflammation, which would be detrimental in NAFLD. Thus, future studies on 11β-HSD1 inhibitors should address their effect on the immune system.

Recent studies have shown that PLIN2 and PLIN3 are involved in the formation of lipid droplets and are involved in the pathophysiology of NAFLD (Carr and Ahima, 2016; Graffmann et al., 2016; Sahini and Borlak, 2016). In particular, PLIN2 has been positively associated with NAFLD (Najt et al., 2016; Orlicky et al., 2019). In this study, we found that HFD can induce PLIN2 up-regulation, while WZS08 can restore it to normal levels (Figure 8). In fact, recent *in vitro* NAFLD models indicate that PLIN2 is associated with NAFLD (Graffmann et al., 2016). Whole-body loss (Libby et al., 2016) or liver-specific loss (Najt et al., 2016) of PLIN2 can prevent HFD-induced obesity, insulin resistance and NAFLD. Therefore, WZS08 can reduce *Plin2* expression to prevent the formation of NAFLD. In fact, WZS08 reduced liver fat content (Figure 6I) and lipid size (Figure 6J). This effect of WZS08 did not result from decreased appetite and intestinal absorption of lipids, because calorie intake (Figure 4D) and fecal lipids (Figure 4C) did not change after the treatment of WZS08. Previous studies also showed that in HFD-induced NAFLD mouse model the expression of *Acs2* was significantly down-regulated (Kim et al., 2004). Indeed, *Acs2* expression and its protein level were significantly reduced in the HFD-induced NAFLD mouse model (Figure 8), while WZS08 (1, 2, and 4 mg/kg) significantly increased its expression (Figure 8).

In conclusion, we synthesized ten benzylidene cyclopentanone derivatives and found that WZS08 was the most potent selective inhibitor of 11β-HSD1 in rats, mice and humans. WZS08 can effectively prevent NAFLD in HFD-induced mouse models.

## Data Availability

The original contributions presented in the study are included in the article/Supplementary Material, further inquiries can be directed to the corresponding author.
