# Supplementary material for: 5-Bis-(2,6-difluoro-benzylidene) Cyclopentanone Acts as a Selective 11β-Hydroxysteroid Dehydrogenase one Inhibitor to Treat Diet-Induced Nonalcoholic Fatty Liver Disease in Mice
Source: Front Pharmacol. 2021 Apr 12;12:594437. doi: 10.3389/fphar.2021.594437 (PMC8072159; doi:10.3389/fphar.2021.594437)
Supplement: Supplementary file 2 [file table1.docx]

### Supplementary Table S1. Primer information

| Primer  Symbol | Gene name | Primer direction | Sequences (5’to 3’) | PCR  (bp) | Accession |
| --- | --- | --- | --- | --- | --- |
| Acs2 | acetyl-CoA synthetase2 | Forward | GGAGCAGAAGGCTGGTAGTG | 300 | NM_153807.2 |
|  |  | Reverse | TCCAGCTCTGCAGGGTAGAT |  |  |
| Ldlr | Low-density lipoprotein receptor | Forward | CAGCTCTGTGTGAACCTGGA | 271 | NM_010700.2 |
|  |  | Reverse | TCAGGGCGCTGTAGATCTTT |  |  |
| Plin2 | perilipin 2 | Forward | CCCTGTCTACCAAGCTCTGC | 246 | AK011014 |
|  |  | Reverse | CAACACAGTGGGACTCATGG |  |  |
| Plin3 | Perilipin 2 | Forward | CTGAGAAAGGCGTCAAGACC | 127 | NM_025836 |
|  |  | Reverse | TTTCTTGAGCCCCAGACACT |  |  |
| Hsd11b1 | [11β-Hydroxysteroid dehydrogenase 1](https://www.baidu.com/link?url=RASn5FVJOHQO5F8yLZuLK2GaE-txBvaDG-Aix0zS1TOK-H6BDM3SQ-dtmZqJCiib&wd=&eqid=adb6e42d00037867000000045854b8a0) | Forward | GGAGCCGCACTTATCTGA | 204 | NM_008288.2 |
|  |  | Reverse | TGCCATTTCTCTTCCAATC |  |  |
| Gapdh | Glyceraldehyde-3-phosphate dehydrogenase | Forward  Reverse | \| ACCCAGAAGACTGTGGATGG \| \| --- \| \| ACACATTGGGGGTAGGAACA \| | 172 | XM_001473623.1 |
